# Do Biliary Stents Affect EUS-Guided Tissue Acquisition (EUS-TA) in Solid Pancreatic Lesions Determining Biliary Obstruction? A Literature Review with Meta-Analysis

**DOI:** 10.3390/cancers15061789

**Published:** 2023-03-15

**Authors:** Antonio Facciorusso, Saurabh Chandan, Paraskevas Gkolfakis, Daryl Ramai, Babu P. Mohan, Andrea Lisotti, Maria Cristina Conti Bellocchi, Ioannis S. Papanikolaou, Benedetto Mangiavillano, Konstantinos Triantafyllou, Eleni Manthopoulou, Ruxandra Mare, Pietro Fusaroli, Stefano Francesco Crinò

**Affiliations:** 1 Gastroenterology Unit, Department of Surgical and Medical Sciences, University of Foggia, 71122 Foggia, Italy; 2 Gastroenterology Unit, CHI Health Creighton University Medical Center, Omaha, NE 68007, USA; 3 Department of Gastroenterology, Hepatopancreatology, and Digestive Oncology, CUB Erasme Hospital, Université Libre de Bruxelles (ULB), 1050 Brussels, Belgium; 4 Gastroenterology and Hepatology, University of Utah Health, Salt Lake City, UT 801385, USA; 5 Gastroenterology Unit, Hospital of Imola, 40026 Imola, Italy; 6 Gastroenterology and Digestive Endoscopy Unit, Department of Medicine, The Pancreas Institute, University Hospital of Verona, 37100 Verona, Italy; 7 Hepatogastroenterology Unit, Second Department of Internal Medicine-Propaedeutic, Medical School, “Attikon” University General Hospital, National and Kapodistrian University of Athens, 10431 Athens, Greece; 8 Gastrointestinal Endoscopy Unit, Humanitas Mater Domini, Via Gerenzano 2, 21053 Castellanza, Italy; 9 Department of Gastroenterology, St. Savvas Oncology Hospital of Athens, 10431 Athens, Greece; 10 Gastroenterology Unit, Department of Internal Medicine II, “Victor Babes” University of Medicine and Pharmacy, 300226 Timisoara, Romania

**Keywords:** EUS, FNA, FNB, ERCP, cancer

## Abstract

**Simple Summary:**

There is a paucity of evidence assessing the impact of bile duct stenting at the time of EUS-guided tissue sampling, either using fine-needle biopsy (EUS-FNB) or fine-needle aspiration (EUS-FNA) in patients with head of pancreas (HOP) masses. Our main aim was to assess the diagnostic accuracy of endoscopic ultrasound-guided tissue acquisition (EUS-TA) in patients with or without concurrent bile duct stents. Pooled accuracy was not statistically different between both patient subsets. However, we found that, compared to plastic stents, patients with biliary metal stents had a significantly lower yield of EUS-TA for pancreatic head lesions.

**Abstract:**

There is a paucity of evidence regarding whether biliary stents influence endoscopic ultrasound-guided tissue acquisition using either fine-needle biopsy (EUS-FNB) or fine-needle aspiration (EUS-FNA), among patients with head of pancreas (HOP) lesions. We aimed at assessing the diagnostic accuracy of endoscopic ultrasound-guided tissue sampling in patients with or without bile duct stents. A total of seven studies with 2458 patients were included. The main aim was to assess overall pooled diagnostic accuracy. A pairwise meta-analysis was performed using a random effects model. Outcomes were expressed as odds ratios (ORs) with 95% confidence intervals (CIs). We found that pooled accuracy was 85.4% (CI 78.8–91.9) and 88.1% (CI 83.3–92.9) in patients with and without stents, respectively. There was no statistically significant difference between the two (OR 0.74; *p* = 0.07). Furthermore, patients with metal stents demonstrated a significant difference (OR 0.54, 0.17–0.97; *p* = 0.05), which was not seen with plastic stents. EUS-FNB showed poorer diagnostic accuracy with concurrent biliary stenting (OR 0.64, 0.43–0.95; *p* = 0.03); however, the same was not observed with EUS-FNA. Compared to plastic stents, metal biliary stenting further impacted the diagnostic accuracy of EUS-guided tissue acquisition for pancreatic head lesions. There was no difference in the rate of procedure-related adverse events between the stent and no-stent groups.

## 1. Introduction

The worldwide incidence of pancreatic cancer has been increasing in recent times. Pancreatic cancer remains one of the more common cancers across males and females, and overall is the seventh leading cause of cancer-related deaths globally. Pancreatic cancer is estimated to soon surpass breast cancer in European countries and is slated to become the third most common cause of cancer-related deaths. A similar trend has already been seen in the United States (U.S.).

Solid lesions in the pancreatic head often present clinically as obstructive jaundice, necessitating the need for ERCP to relieve the obstruction. On the other hand, a diagnostic evaluation is typically performed using EUS-TA. EUS-TA, with the use of fine-needle aspiration (FNA) and/or biopsy (FNB), is frequently employed as the gold standard intervention for obtaining tissue for the purposes of establishing a cytologic or histologic diagnosis of pancreatic malignancies. While solid pancreatic lesions may have both malignant and non-malignant causes, it is important to note that pancreatic adenocarcinoma is by far the most common malignant cause and is found in around 85% of solid pancreatic lesions. Furthermore, only 1/5th of patients with this serious etiology have lesions that may be surgically resectable at the time of presentation. In recent times, outcomes for patients with pancreatic lesions considered to be resectable, borderline resectable and locally advanced tumors have been investigated by multiple studies. Despite this, the performance of EUS in accurately assessing a lesion’s candidacy for resection as well as differentiating between resectable and borderline resectable or locally advanced lesions is paramount due to the differences in potential therapeutic modalities. The growing use of neoadjuvant chemotherapy in patients with potentially resectable pancreatic cancers also requires histological confirmation prior to initiating any treatments. Regardless, the decision regarding tissue acquisition for establishing diagnosis tends to vary across practice patterns and is often based on several patient factors and characteristics.

Early tumor detection is key to significantly impacting the prognosis and morbidity among patients with pancreatic cancer. This means prompt detection of potentially resectable tumors should be undertaken by use of cross-sectional imaging modalities, including computed tomography (CT), magnetic resonance imaging (MRI), positron emission tomography (PET) as well as EUS-TA either through EUS-FNA and/or EUS-FNB. While there are several perceived advantages to performing EUS before ERCP, tumor staging is generally thought to be more accurate in patients without biliary endoprosthesis. Factors such as the underlying pathology as well as disease stage, localized or advanced, guide appropriate stent selection for biliary interventions. Data have shown that EUS-FNA has higher sensitivity and diagnostic accuracy than ERCP tissue sampling in patients with pancreatic malignancies. The reported overall sensitivity and accuracy for EUS-FNA are reported to be over 90% compared to about 50–53% for ERCP-based sampling, respectively [1].

Tissue sampling using endoscopic ultrasound, also termed EUS-TA, is the gold standard procedure for the characterization of solid pancreatic lesions (SPLs). However, the diagnostic accuracy of EUS-TA is highly variable due to several factors, including operator variability, needle type and caliper [1,2], use of contrast enhancement [3], final diagnosis [4,5] or whether rapid on-site evaluation (ROSE) is present or not [6,7].

Solid lesions located in the head of the pancreas (SPLs) often present clinically with jaundice. However, by the time SPLs are diagnosed, the majority of lesions are at an advanced stage and hence, unresectable. As a corollary, prompt biliary drainage by means of stent placement through endoscopic retrograde cholangiopancreatography (ERCP) is often required for palliation and prevention of cholangitis.

Given the concurrent need for cyto/histological confirmation that is mandatory before chemotherapy, performing both the EUS and ERCP procedures at the same time, might represent a valuable option. Moreover, ERCP is more widely available than EUS, and relieving bile duct obstruction is considered more clinically urgent than tissue acquisition for establishing a pathological diagnosis [8]. Therefore, in most instances, biliary stenting frequently precedes EUS-TA. It must also be noted that ERCP can be associated with some sinister and potentially life-threatening complications due to inadvertent cannulation of the pancreatic duct. Post-ERCP pancreatitis has been reported to occur in about 5% of ERCP procedures, but among patients with higher morbidity, such as in patients with bile duct malignancy, the risk of post-ERCP pancreatitis can be higher.

Historically, EUS-guided fine-needle aspiration (EUS-FNA) using needles with simple tip bevels was performed for EUS-TA, with reported diagnostic accuracy ranging from 87 to 92%. However, its poorer performance for gastrointestinal wall lesions and inability to preserve architecture remained an area of concern. Over the past few years, the development of biopsy needles with beveled side slots (Quick-Core^®^ and ProCore^®^, Cook Medical, Bloomington, IN, USA) have been increasingly utilized for EUS-TA. In order to improve the accuracy of EUS-TA, the beveled side slot design has been further altered to be forward-facing (20G ProCore^®^, Cook Medical, Bloomington, IN, USA). While the published literature among prospective studies evaluating various EUS-TA techniques for solid pancreatic lesions is conflicting, taken cumulatively, both techniques have shown comparable and high diagnostic accuracy and specimen adequacy for accurate diagnosis. EUS-TA has also been shown to be a clinically safe intervention with reported adverse events (AE) being relatively rare. Studies have shown that the risk of pancreatitis following EUS-TA, bleeding, and perforation events is estimated to be in 0.44, 0.10 and 0.02% of cases, respectively. A recent network meta-analysis assessing EUS-guided tissue acquisition techniques in patients with solid pancreatic lesions was performed, including 27 randomized controlled trials with 2711 patients. The authors found that no particular sampling technique was better, based on needle type (FNA vs. FNB) or needle size, whether 19-, 22- or 25-gauge. However, these findings were backed by an overall low quality of evidence. Additionally, they also showed that when comparing outcomes using 25- or 22-gauge FNA needles, or 22-gauge FNB and 22-gauge FNA needles, there was no difference in terms of the diagnostic accuracy; adequacy of tissue samples and histology; relative risk (RR), 1.03; 95% confidence interval (CI), 0.91–1.17 and RR, 1.03; and 95% CI, 0.89–1.18, respectively. These results were further validated when taking into consideration only those studies without the use of rapid on-site cytologic evaluation (ROSE) and the fanning technique.

There is conflicting evidence regarding the impact of biliary stents on the diagnostic accuracy of EUS-TA. This is likely due to poor visualization of the lesion from acoustic shadowing, reverberation and/or surrounding inflammation associated with the stent [9,10,11]. Additionally, these studies seem to suggest that plastic or self-expandable metal stents (SEMSs) could have a dissimilar effect because of differences in their material and diameter. While previously published reports have suggested that the presence of biliary plastic stents or SEMSs does not influence the diagnostic accuracy of EUS-guided fine-needle aspiration (EUS-FNA) in pancreatic head lesions [9,10,11,12], Kim et al. reported a decrease in the accuracy of EUS-FNA with concomitant stents, regardless of their type [13]. The negative impact of biliary stents on EUS-guided fine-needle biopsy (FNB) has been further confirmed in a recent large multicenter Italian series [11]. Another recent retrospective analysis by Oppong et al. [14] analyzed the ability of EUS to diagnose pancreatic lesions for suitability in terms of surgical resection based on whether a bile duct stent was present or not. Out of the 90 patients included in the analysis, over 50% of patients had a concomitant bile duct stent at the time of EUS-TA. Among these, 36 stents were plastic, and 13 were metal stents. The authors reported that overall, 20 patients underwent successful surgical resection. Furthermore, while disease staging was successful in all patients without biliary stents, it was only possible in 97% of patients with plastic stents and a mere 54% of patients with metal bile duct stents [14].

Since supportive evidence showing the negative impact of biliary stents on EUS-TA may influence the choice or sequence of procedures, as well as the type of stent (plastic versus SEMS), to be placed during ERCP, a thorough review of the published literature is needed to inform clinical practice and forthcoming guidelines. Therefore, we performed a systematic review following the PRISMA methodology (Supplementary Table S1) and a meta-analysis of studies comparing the diagnostic accuracy of EUS-TA before and after biliary stent placement in jaundiced patients with pancreatic head masses.

## 2. Materials and Methods

### 2.1. Selection Criteria

Randomized controlled trials (RCTs) and/or observational cohort studies meeting the following inclusion criteria were included: (a) patients > 18 years with solid pancreatic head masses and jaundice; (b) intervention: EUS-guided tissue sampling through side-fenestrated FNB (ProCore^®^, Cook Medical Inc., Bloomington, IN, USA), end-cutting FNB (Franseen needle (Acquire^®^, Boston Scientific, Marlborough, MA, USA) or Fork-tip needle (SharkCore^®^, Medtronic, Dublin, Ireland)) or FNA in patients with plastic or metal biliary stents; (c) comparator: EUS-TA through side-fenestrated FNB, end-cutting FNB or FNA in patients without biliary stents; and (d) outcomes: primary outcome was diagnostic accuracy, whereas secondary outcomes were overall pooled sample adequacy, diagnostic sensitivity, specificity and mean number of needle passes. Additionally, safety data, including adverse events, were also analyzed.

Our exclusion criteria were as follows: (a) single-arm cohort studies without a comparator, (b) studies not reporting subgroup analysis restricted to patients with biliary stents, and (c) studies not reporting our primary and secondary outcomes of interest.

### 2.2. Search Strategy

A detailed database search was independently performed by two authors (AF and PG) on PubMed/Medline and Embase for all published literature of interest through March 2022 using the following keywords: ((((endoscopic ultrasound [MeSH Terms]) OR (EUS [MeSH Terms])) AND (biopsy [MeSH Terms])) OR (aspiration [MeSH Terms])) AND (stent). There was no language restriction.

A supplemental manual search was conducted on additional databases (Google Scholar and Cochrane Library) as well as by cross-examining the references of all the main review articles on this topic to identify any additional studies. In cases of potential overlap among publications from the same authors/patient population, the most recent and full-length articles were included. We excluded conference abstracts during our literature review. Data were extracted by two authors (AF and PG) into a previously designed Excel sheet. Data variables included study authors, publication date, study country, study design, age, sex, presence or absence of biliary stent, type of stent, needle caliper, time between the ERCP and EUS and whether ROSE was available at the time of EUS-TA or not.

Quality assessment was independently performed by two authors (AF and SFC) according to the Newcastle–Ottawa scale for non-randomized studies [15]. All disagreements were addressed with re-evaluation by a third author independently (PF). In situations where a consensus could not be reached, overlapping studies were included in the final analysis, and any potential effects were assessed using sensitivity analysis of the pooled outcomes by leaving out one study at a time.

### 2.3. Outcomes

The primary outcome assessed was the overall diagnostic accuracy, defined as the summary of true positives (TPs) + true negatives (TNs) divided by the total number of patients. Surgery or the evolution of disease assessed (for at least 6 months) by a combination of clinical course and/or imaging studies was considered the gold standard for establishing diagnosis [16]. Our secondary outcomes were overall pooled adequacy, defined by the authors as proportion of lesions sampled in which the obtained material is representative of the target site and sufficient for diagnosis [16]; pooled diagnostic sensitivity, calculated as the proportion of positives identified with the test (TPs) on the prevalence of disease in the study cohort (TPs + false negatives (FNs)); pooled diagnostic specificity, calculated as the proportion of negatives correctly identified as such (TNs) among the patients who were not affected by the disease in the study cohort (TNs + false positives (FPs)); number of needle passes required to obtain adequate sample; and overall adverse events.

### 2.4. Statistical Analysis

Statistical analytic techniques were performed to assess the overall pooled outcomes along with 95% confidence intervals (CIs). Random effects model was utilized for all of our outcomes, as previously validated by DerSimonian and Laird. We reported our outcomes as pooled odds ratios (ORs) or mean differences and 95% CIs, when applicable.

Heterogeneity in our summary estimates was calculated through I² tests, with I^2^ < 30% interpreted as low-level heterogeneity and I^2^ between 30 and 60% as moderate heterogeneity [17].

All study outcomes were assessed in the overall cohort and the two subgroups based on the use of plastic stents versus SEMSs. Sensitivity analyses in the context of the primary outcome were also based on needle used (FNB versus FNA) and availability of ROSE.

Adverse events were analyzed and reported descriptively, as definitions varied across the studies.

All statistical analyses were performed using RevMan version 5 from the Cochrane collaboration. For meta-analysis, a two-tailed *p*-value of less than 0.05 was considered statistically significant.

## 3. Results

### 3.1. Included Studies

**Figure 1:**
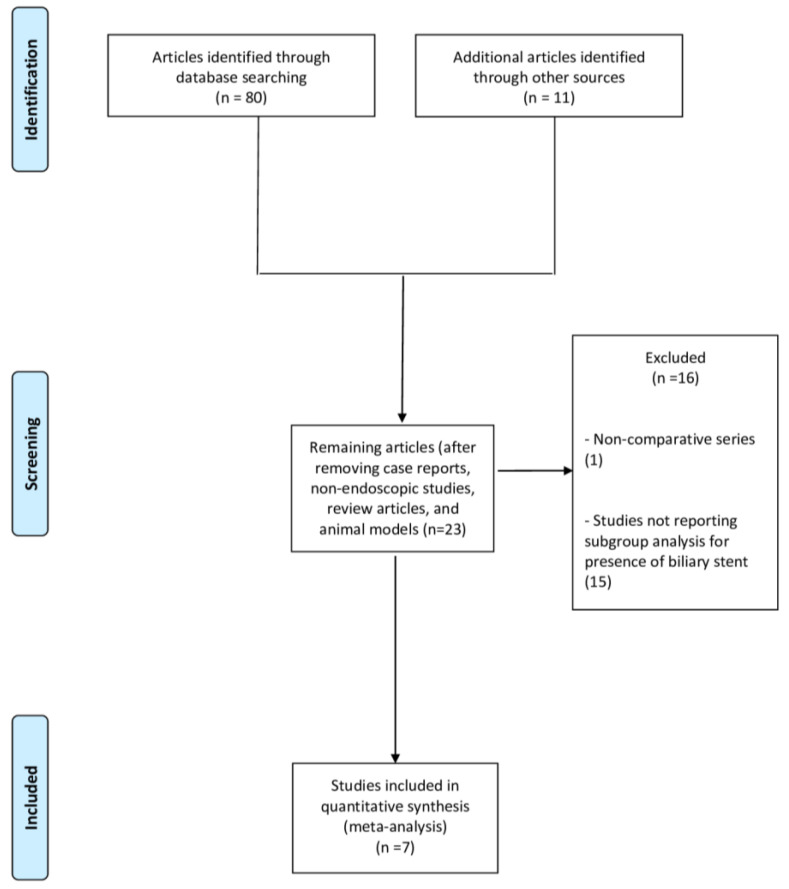
Flow chart of the included studies.

From 91 studies identified using the search strategy, we included seven retrospective case-control studies [10,11,12,13,18,19,20] (Figure 1) with 2458 patients.

Of note, the study by Siddiqui et al. was excluded as it included only patients with biliary stents [9]. Patient characteristics and demographics are summarized in Table 1.

Four studies were conducted in Europe [10,11,18,20] and three in the USA [12,13,19]. Patients were recruited between periods ranging from 1998 to 2020. There was a similarity between the two study cohorts in terms of baseline demographic and lesion characteristics. Overall, the majority of participants were males, and the mean age was 69 years. The mean lesion size ranged from 29 to 35 mm, and most of the biliary stents used were made of plastic. In particular, the study by Antonini et al. compared only patients with plastic stents versus patients without stents [18]. Two studies included patients with FNA [12,19], two studies included patients with FNB [11,18], while the other studies included patients who underwent EUS-FNB or FNA. The most common needle caliber was 22G, both with FNB and FNA. ROSE was available for the majority of patients in the three studies [12,13,19].

All included studies were assessed for quality, and the summary of these findings is outlined in Supplementary Table S2. Overall, we found that four studies [10,11,12,18] showed a low risk of bias, whereas other studies showed a higher risk of outcome reporting bias as well as selection bias.

### 3.2. Diagnostic Accuracy

**Figure 2:**
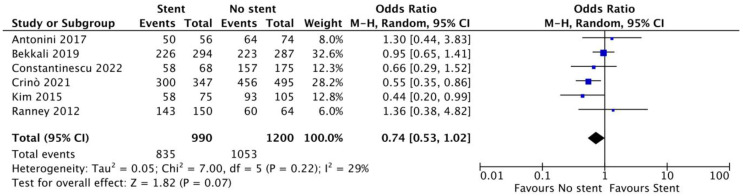
Forest plot—pooled diagnostic accuracy. References [10,11,12,13,18,20].

Based on six studies [10,11,12,13,18,20] (990 patients with biliary stents and 1200 without stents), the overall pooled diagnostic accuracy was 85.4% (95% CI 78.8–91.9%) and 88.1% (83.3–92.9%) in patients with and without biliary stenting, respectively. There was no significant difference between the two approaches (OR 0.74, 95% CI 0.53–1.02; *p* = 0.07). Overall, heterogeneity was low (I^2^ = 29%; Figure 2).

As reported in Table 2, no difference was observed between patients with plastic stents versus no stents (OR 0.89, 0.51–1.54; *p* = 0.67), whereas a significant difference was observed in the comparison between patients with SEMSs versus those without SEMSs (OR 0.54, 0.17–0.97; *p* = 0.05). In particular, pooled accuracy was 87.9% (82.6–93.1%) in patients with plastic stents and 80.9% (63.9–84.2%) in patients with SEMSs.

Sensitivity analyses restricted to studies with FNA confirmed that there was no statistically significant difference between the two groups (OR 1.36, 0.38–4.82; *p* = 0.63). We also found that the overall pooled diagnostic accuracy with EUS-FNB was significantly lower in patients who had bile duct stents (OR 0.64, 0.43–0.95; *p* = 0.03). The availability of ROSE did not influence the results of the main analysis (OR 0.69, 0.23–2.06; *p* = 0.51). The heterogeneity of the sensitivity analyses was mainly low (Table 2).

### 3.3. Secondary Outcomes

**Figure 3:**
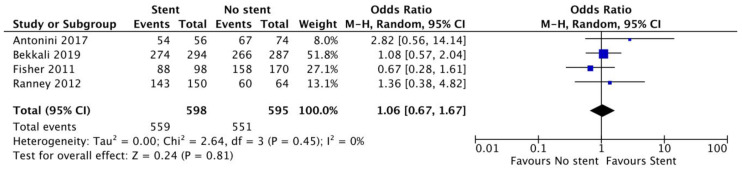
Forest plot of sample adequacy analysis. No difference in terms of sample adequacy was observed between patients with biliary stents and those without stents (OR 1.06, 0.67–1.67; *p* = 0.81, I^2^ = 0%). References [10,12,18,19].

Assessing data from four studies [10,12,18,19], we found no difference in terms of sample adequacy between the two groups (OR 1.06, 0.67–1.67; *p* = 0.81, I^2^ = 0%, Figure 3). These findings are further summarized in Table 3. Overall sample adequacy was 94% (91.8–96.2%) in patients with biliary stents and 92.7% (90.6–94.8%) in patients without stents. No difference between the two groups was observed: plastic stents (OR 1.35, 0.71–2.55; *p* = 0.36) and SEMSs (OR 1.10, 0.55–2.20; *p* = 0.79).

Based on five studies [10,11,12,13,18], overall diagnostic sensitivity among patients with bile duct stents was significantly lower (OR 0.59, 0.44–0.80; *p* < 0.001, I^2^ = 14%) with pooled rates of 82.9% (72.8–93%) and 87.5% (81.7–93.3%) in the two groups, respectively (Supplementary Figure S1). This significant difference was confirmed in the subgroup of studies with SEMSs (OR 0.61, 0.43–0.86; *p* = 0.006; Supplementary Figure S2b), whereas no difference was observed in the subgroup with plastic stents (OR 0.68, 0.42–1.10; *p* = 0.12; Supplementary Figure S2a).

The number of needle passes required for the acquisition of diagnostic samples was not significantly different between the two groups (mean difference −0.09, −0.30 to 0.11; *p* = 0.38; I^2^ = 86%; Supplementary Figure S3).

In terms of complication rates, two studies reported no procedure-related adverse events in the stent vs. no-stent cohorts. In one of these studies, only plastic biliary stents were placed, whereas in the other study, a combination of plastic stents and biliary SEMSs were utilized. Only the study by Fisher et al. [19], which utilized a combination of plastic stents and biliary SEMSs, reported procedure-related adverse events with no difference between the stent vs. no-stent groups in terms of post-procedure pancreatitis, self-limited GI bleeding, perforation events and bile leak. Overall, there was no statistically significant difference between the two groups (4% vs. 4.7%, *p* = 0.75). Details of the complications as reported by the included studies are reported in Supplementary Table S3.

## 4. Discussion

The body of evidence on whether the presence of biliary stents influences the diagnostic accuracy of EUS-TA is limited overall, with conflicting results reported by different consensus groups. Patients presenting with biliary obstruction and solid pancreatic lesions often undergo endoscopic retrograde cholangiopancreatography (ERCP) at the initial stages of management, especially in areas where the ability to perform EUS is not widely available. In these cases, EUS analysis is preceded by successful biliary drainage. The Canadian Society for Endoscopic Ultrasound states that EUS-TA should precede ERCP. On the other hand, the international consensus for the endoscopic management of distal biliary strictures states that ERCP for relieving bile duct obstruction does not influence outcomes of EUS-TA. They further outline that performing ERCP first is appropriate for both diagnostic and therapeutic purposes in these patients [21,22]. Decompression via ERCP and biliary stenting often results in correcting underlying jaundice as well as provides relief from cholestatic pruritus. Bile duct stenting can also facilitate patients in beginning their chemotherapeutic interventions by offsetting the risk of cholestasis-related chemotoxicity. Biliary endotherapy is a commonly performed ERCP intervention, with a technical success rate in over 90% of cases and has thus become the most frequently performed intervention relieving biliary obstruction. Whether a plastic or metallic biliary stent is placed at the time of ERCP largely depends on factors such as the patient’s overall prognosis, the certainty of diagnosis and planned chemo- or radiation-based interventions, as well as the cost-effectiveness and operator expertise. In recent times, the use of metallic biliary stents has become increasingly more common in clinical practice for the treatment of both benign and malignant biliary strictures. Although the use of metallic bile duct stents was initially discouraged by pancreatic surgeons due to perceived concerns of increasing the difficulty of resection, more recent data suggest that metallic bile duct stents do not interfere with planned surgical interventions, such as pancreaticoduodenectomy, as long as the stent is not involving the hilum.

Our analysis is the first and most comprehensive review and meta-analysis on this topic, allowing us to report several key findings. First, diagnostic accuracy in patients with biliary stents was 85.4% versus 88.1% in patients without stents (OR 0.74, 95% CI 0.53–1.02; *p* = 0.07); therefore, only a non-significant trend towards higher accuracy rates in patients without stents was observed. No difference was seen among patients with plastic stents versus no stents (*p* = 0.67), while a statistically significant difference was seen when comparing patients with and without metal stents (*p* = 0.05). It must also be noted that plastic stents or SEMSs may have different effects on the outcomes of EUS-TA due to the difference in material as well as the diameter (plastic stents are usually 10 Fr, whereas SEMSs are generally 10 mm). Furthermore, a comparative analysis based on the sizes of biliary stents used was outside the scope of our study.

Our results are in concordance with the findings of a recent English study [10], which demonstrated evidence of a greater impact of SEMSs on the diagnostic accuracy of EUS-TA. Another recent study assessed the impact of biliary stents on EUS-TA and found that among patients undergoing pancreaticoduodenectomy for suspected pancreatic cancer, over half of whom had a concurrent bile duct stent, EUS-based disease staging was significantly affected in the presence of metallic bile duct stents compared to plastic stents or the absence of bile duct stents. These findings are of high importance, considering that several studies have reported that SEMSs are superior to plastic stents among patients with distal biliary strictures for the purposes of preoperative biliary drainage and palliation of obstruction [23,24,25]. Consequently, SEMSs are increasingly being used over plastic stents for this indication. Moreover, fully covered SEMSs can be used even before cyto-histological confirmation of malignancy because of their removability. On the other hand, a larger caliber of SEMSs as well as the acoustic shadowing might be responsible for the lower accuracy and sensitivity rates reported. Additionally, the reverberation artifacts that are observed with the use of SEMSs could completely or partially mask small lesions, making the sampling of these difficult. This point could be even more relevant in low-volume or less experienced centers. On the other hand, prior studies [12,19] have shown conflicting results in this regard; of note, these studies used mainly EUS-FNA with ROSE, whereas our meta-analysis showed no difference between these two groups.

In the current study, sensitivity analysis of the studies with EUS-FNA demonstrated a non-significant difference between patients with or without a biliary stent. However, biliary stents significantly reduced the diagnostic accuracy of EUS-FNB. There are at least two explanations for this finding. First, it is likely that the diagnostic accuracy of EUS-FNA was already low, even without biliary stents. As a corollary, the placement of a biliary stent may further negatively impact diagnostic accuracy. Second, EUS-FNB is a more recent technique compared with EUS-FNA. Therefore, it is possible that a larger number of patients who underwent EUS-FNB underwent biliary drainage using SEMSs due to evidence demonstrating their superiority over plastic stents [26]. In other words, both EUS-FNB and SEMSs have become widespread during recent decades [27,28,29,30], and it is possible that the negative impact of biliary stents was related to the more frequent use of SEMSs rather than plastic stents.

The sample adequacy was similar between the two groups and not dependent on the type of stent used. Probably, torquing or changing to the long position of the echoendoscope might provide a better window for tissue sampling, and the needle could pass through the meshes of SEMSs. This could increase the chances of obtaining adequate samples and affect final accuracy, as shown above, and in previous studies [18].

Finally, both the total number of needle passes required to obtain adequate diagnostic samples as well as the overall procedure-related adverse event rate were not significantly different between the two groups ( *p* = 0.38 and *p* = 0.75, respectively). It is important to note that adverse events were reported by only three of the seven studies included in our analysis, which included a combination of plastic stents and biliary SEMSs. While two of these studies reported no procedure-related adverse events in either group, another study reported similar rates of post-procedure pancreatitis, GI bleeding, perforation events and bile leaks between the stent and no-stent cohorts. Despite this, the overall pooled rate of procedure-related adverse events was low, and we conclude that the presence of a biliary stent does not increase the rate of complications of these procedures.

Our analysis also has several limitations, most of which are inherent to any meta-analysis. First, the number of included studies and recruited patients were relatively limited, and the evidence was based only on retrospective series. This may have contributed to the selection and reporting bias. Consequently, several sensitivity analyses were conducted to assess all the potential confounders in the analysis, and we found low heterogeneity in all our outcomes. Second, we were unable to perform subgroup analysis based on needle caliber or assess outcomes with newer end-cutting FNB needles due to the lack of data. Likewise, the impact of the timing of ERCP before EUS-TA was not assessed due to the lack of data. However, a recent Italian multicenter study [11] suggested a decreasing trend of diagnostic accuracy as time elapses from ERCP with biliary stent placements. Third, several of our outcomes were assessed from a limited number of studies and, as a result, may not be generalizable. Moreover, several potential confounders, such as the type of suction, type of fixation, ROSE expertise and the number of passes could not be assessed due to the lack of subgroup data in the included studies. Other potential limitation could be the large timespan of recruitment period in the included studies with the use of different equipment and needles. However, we performed a sensitivity analysis based on the needles used (whether older FNA needles or FNB). Finally, the impact of the size of the lesion was not studied, but it is likely that the negative impact of biliary stents on diagnostic accuracy is more evident in the case of smaller lesions, as suggested in previous studies [10,11].

## 5. Conclusions

In conclusion, despite certain limitations, our comprehensive review and meta-analysis suggest a negative impact of the presence of a biliary stent on the diagnostic accuracy of EUS-TA in solid pancreatic lesions. This is particularly significant in the case of biliary endotherapy with SEMSs, while we found no significant difference with plastic biliary stents. Additionally, we found no difference in the overall rate of EUS-related adverse events among patients with or without biliary stents. Therefore, in jaundiced patients with pancreatic head lesions, EUS-TA should precede ERCP, especially when SEMSs are being employed.

## Figures and Tables

**Table 1:** Characteristics of included studies.

Study (Country) Period/Design	Study Group	Age	Gender Male	Lesion Size (mm)	Type of Stent (Plastic/Metal)	Needle	Needle Caliper and Time between ERCP and EUS	ROSE
Crinò, 2021 [11] Italy 2017–2019/ Retrospective	Stent: 347 No stent: 495	68 (57.5–76) 70 (64–78)	202 (58.2%) 271 (58.8%)	29.3 ± 8.9 31.7 ± 8.7	217 (62.5%)/130 (37.5%)	Side-fenestrated FNB 335/end-cutting FNB 507	101(29.1%) 25G/217(62.5%) 22G/29(8.4%) 20G 129 (26.1%) 25G/294 (59.4%) 22G/72 (14.5%) 20G 11 days (3–30)	41 (11.8%) 70 (14.1%)
Antonini, 2017 [18] Italy 2013–2015/Retrospective	Stent: 56 No stent: 74	69 ± 9.9 70.3 ± 10.3	33 (58.9%) 46 (62.2%)	30.7 ± 12.6 30.8 ± 8.5	56 (100%)/0 (0%)	Side-fenestrated FNB 100%	29 (51.8%) 22G/27 (48.2%) 25G 29 (39.2%) 22G/45 (60.8%) 25G <48 h: 10 (17.8%)	16 (28.5%) 14 (18.9%)
Bekkali, 2018 [10] UK 2010–2016/Retrospective	Stent: 294 No stent: 287	66.3 ± 9.4 65 ± 11.4	160 (54.4%) 185 (64.4%)	35 (25–40) 31 (25–39)	137 (46.6%)/157 (53.4%)	290 (41.5%) FNA/228 (32.7%) side-fenestrated FNB/179 (25.6%) Fork-tip FNB	382 (54.7%) 22G/269 (38.5%) 25G NR 22G, 25G FNA	64 (21.7%) 49 (13.3%)
Fisher, 2011 [19] USA 1998–2009/Retrospective	Stent: 98 No stent: 170	69.2 68.2	44.8% 44.7%	32.3 33.4	66 (67.3%)/6 (6.1%)	FNA	77% 22G/11.5% 25G 54.1% 22G/34.7% 25G <1 day 11 (11.3%)	100% 100%
Kim, 2015 [13] USA 2005–2013/Retrospective	Stent: 75 No stent: 105	65 ± 12	108 (60%)	>3 cm: 76 (42%)	64 (85.3%)/11 (14.7%)	75 (42%) FNA/105 (58%) side-fenestrated FNB	NR 18 days (1–247)	81 (45%)
Ranney, 2012 [12] USA 2006–2010/Retrospective	Stent: 150 No stent: 64	68 (58–75) 69 (63–78)	105 (49%) 32 (50%)	30 (21–30) 30 (25–30)	105 (70%)/45 (30%)	FNA	NR	100% 100%
Constantinescu, 2022 [20] Romania 2016–2020/Retrospective	Stent: 68 No stent: 175	62.6 ± 12.23 61.89 ± 12.83	40 (58.8%) 98 (56%)	<2 cm: 9 (13.2%) <2 cm: 17 (7%)	58 (85.3%)/10 (14.7%)	FNA or side-fenestrated FNB or Franseen FNB	22G NR	0% 0%

Data are reported as absolute numbers (percentages) or mean (±standard deviation or with range). Abbreviations: FNA, fine-needle aspiration; FNB, fine-needle biopsy; NR, not reported; ROSE, rapid on-site cytologic evaluation.

**Table 2:** Sensitivity analysis concerning the primary outcome (diagnostic accuracy).

Variable	Subgroup	No. of Studies	No. of Patients	Odds Ratio (95% CI) *p*-Value	Within-Group Heterogeneity (I^2^)
Type of stent	Plastic	5	Stent: 573 No stent: 1095	0.89 (0.51–1.54) 0.67	21%
	Metal	4	Stent: 342 No stent: 1021	0.54 (0.17–0.97) 0.05	17%
Needle	FNB	3	Stent:471 No stent: 744	0.64 (0.43–0.95) 0.03	7%
	FNA	1	Stent: 150 No stent: 64	1.36 (0.38–4.82) 0.63	NA
Availability of ROSE	Yes	2	Stent: 225 No stent: 169	0.69 (0.23–2.06) 0.51	34%
Mean number of needle passes	2	2	Stent: 218 No stent: 239	0.82 (0.41–1.65) 0.59	0%
	>2	3	Stent: 478 No stent: 674	0.80 (0.67–1.82) 0.63	25%

Abbreviation: CI, confidence interval; FNA, fine-needle aspiration; FNB, fine-needle biopsy; NA, not applicable; ROSE, rapid on-site cytologic evaluation.

**Table 3:** Secondary outcomes.

Overall Study Sample				
Outcome	No. of Studies	No. of Patients	Odds Ratio (95% CI)	Within-Group Heterogeneity (I^2^)
Sample adequacy	4	Stent: 598 No stent: 595	1.06 (0.67–1.67) *p* = 0.81	0%
Diagnostic sensitivity	5	Stent: 922 No stent: 1025	0.59 (0.44–0.80) *p* < 0.001	14%
**Outcome**	**No. of Studies**	**No. of patients**	**Mean difference (95% CI)**	**Within-group heterogeneity (I^2^)**
Number of needle passes	6	Stent: 794 No stent: 1083	−0.09 (−0.30 to 0.11) *p* = 0.38	86%
**Plastic stent**				
**Outcome**	**No. of Studies**	**No. of patients**	**Odds ratio (95% CI)**	**Within-group heterogeneity (I^2^)**
Sample adequacy	3	Stent: 298 No stent: 425	1.35 (0.71–2.55) *p* = 0.36	0%
Diagnostic sensitivity	4	Stent: 515 No stent: 920	0.68 (0.42–1.10) *p* = 0.12	45%
**Metal stent**				
**Outcome**	**No. of Studies**	**No. of patients**	**Odds ratio (95% CI)**	**Within-group heterogeneity (I^2^)**
Sample adequacy	2	Stent: 202 No stent: 351	1.10 (0.55–2.20) *p* = 0.79	0%
Diagnostic sensitivity	3	Stent: 332 No stent: 846	0.61 (0.43–0.86) *p* = 0.006	0%

## Data Availability

The data can be shared up on request.

## Supplementary Materials

The following supporting information can be downloaded at: https://www.mdpi.com/article/10.3390/cancers15061789/s1, Figure S1: Forest plot of diagnostic sensitivity analysis; Figure S2: Forest plot comparing diagnostic sensitivity in the subgroup of patients with (a) plastic stents and (b) metal stents; Figure S3: Forest plot comparing the number of needle passes between patients with biliary stents versus patients without stents. Table S1: PRISMA checklist. Table S2: Risk of bias assessment and quality of included studies. Table S3: Adverse events reported in the included studies.
