# Supplementary material for: Do Biliary Stents Affect EUS-Guided Tissue Acquisition (EUS-TA) in Solid Pancreatic Lesions Determining Biliary Obstruction? A Literature Review with Meta-Analysis
Source: Cancers (Basel). 2023 Mar 15;15(6):1789. doi: 10.3390/cancers15061789 (PMC10046620; doi:10.3390/cancers15061789)

ONLINE SUPPLEMENT

Supplementary Table S1. PRISMA

Section/topic	#	Checklist item	Reported on page #
**TITLE**			
Title	1	Identify the report as a systematic review, meta-analysis, or both.	1
**ABSTRACT**			
Structured summary	2	Provide a structured summary including, as applicable: background; objectives; data sources; study eligibility criteria, participants, and interventions; study appraisal and synthesis methods; results; limitations; conclusions and implications of key findings; systematic review registration number.	2
**INTRODUCTION**			
Rationale	3	Describe the rationale for the review in the context of what is already known.	4
Objectives	4	Provide an explicit statement of questions being addressed with reference to participants, interventions, comparisons, outcomes, and study design (PICOS).	4
**METHODS**			
Protocol and registration	5	Indicate if a review protocol exists, if and where it can be accessed (e.g., Web address), and, if available, provide registration information including registration number.	NA
Eligibility criteria	6	Specify study characteristics (e.g., PICOS, length of follow-up) and report characteristics (e.g., years considered, language, publication status) used as criteria for eligibility, giving rationale.	5
Information sources	7	Describe all information sources (e.g., databases with dates of coverage, contact with study authors to identify additional studies) in the search and date last searched.	5
Search	8	Present full electronic search strategy for at least one database, including any limits used, such that it could be repeated.	5
Study selection	9	State the process for selecting studies (i.e., screening, eligibility, included in systematic review, and, if applicable, included in the meta-analysis).	5-6

Data collection process	10	Describe method of data extraction from reports (e.g., piloted forms, independently, in duplicate) and any processes for obtaining and confirming data from investigators.	5-6
Data items	11	List and define all variables for which data were sought (e.g., PICOS, funding sources) and any assumptions and simplifications made.	5-6
Risk of bias in individual studies	12	Describe methods used for assessing risk of bias of individual studies (including specification of whether this was done at the study or outcome level), and how this information is to be used in any data synthesis.	6-7
Summary measures	13	State the principal summary measures (e.g., risk ratio, difference in means).	6-7
Synthesis of results	14	Describe the methods of handling data and combining results of studies, if done, including measures of consistency (e.g., I^2) for each meta-analysis.	6-7

Section/topic	#	Checklist item	Reported on page #
Risk of bias across studies	15	Specify any assessment of risk of bias that may affect the cumulative evidence (e.g., publication bias, selective reporting within studies).	7
Additional analyses	16	Describe methods of additional analyses (e.g., sensitivity or subgroup analyses, meta-regression), if done, indicating which were pre-specified.	7
**RESULTS**			
Study selection	17	Give numbers of studies screened, assessed for eligibility, and included in the review, with reasons for exclusions at each stage, ideally with a flow diagram.	7
Study characteristics	18	For each study, present characteristics for which data were extracted (e.g., study size, PICOS, follow-up period) and provide the citations.	7
Risk of bias within studies	19	Present data on risk of bias of each study and, if available, any outcome level assessment (see item 12).	7-9
Results of individual studies	20	For all outcomes considered (benefits or harms), present, for each study: (a) simple summary data for each intervention group (b) effect estimates and confidence intervals, ideally with a forest plot.	7-9
Synthesis of results	21	Present results of each meta-analysis done, including confidence intervals and measures of consistency.	7-9
Risk of bias across studies	22	Present results of any assessment of risk of bias across studies (see Item 15).	7-10

Additional analysis	23	Give results of additional analyses, if done (e.g., sensitivity or subgroup analyses, meta-regression [see Item 16]).	10-11
**DISCUSSION**			
Summary of evidence	24	Summarize the main findings including the strength of evidence for each main outcome; consider their relevance to key groups (e.g., healthcare providers, users, and policy makers).	12-13
Limitations	25	Discuss limitations at study and outcome level (e.g., risk of bias), and at review-level (e.g., incomplete retrieval of identified research, reporting bias).	13-14
Conclusions	26	Provide a general interpretation of the results in the context of other evidence, and implications for future research.	14
**FUNDING**			
Funding	27	Describe sources of funding for the systematic review and other support (e.g., supply of data); role of funders for the systematic review.	1

Supplementary Table S2. Risk of bias assessment and quality of included studies.

Observational studies				
	Selection	Comparability	Outcome	Overall quality
Crinò 2021	***	**	***	H
Antonini 2017	***	**	***	H
Bekkali 2018	***	**	***	H
Fisher 2011	**	**	*	L
Kim 2015	**	**	**	L
Ranney 2012	***	**	***	H
Constantinescu 2022	***	**	*	L

L, low; H, high; U, unclear; M, moderate.
Study quality assessment performed by means of Newcastle/Ottawa scale (each asterisk represents if the respective criterion within the subsection was satisfied)

Supplementary Table S3. Adverse events reported in the included studies

Study	Stent	No stent
Crinò 2021	None	None
Antonini 2017	None	None
Bekkali 2018	None	None
Fisher 2011	Pancreatitis 2 (2%)<br>Bleeding 1 (1%)<br>Perforation 1 (1%)	Pancreatitis 4 (2.4%)<br>Perforation 1 (0.6%)<br>Bile leak 1 (0.6%)
Kim 2015	None	None

Ranney 2012	None	None
Constantinescu 2022	None	None

Supplementary Figure S1. Forest plot of diagnostic sensitivity analysis

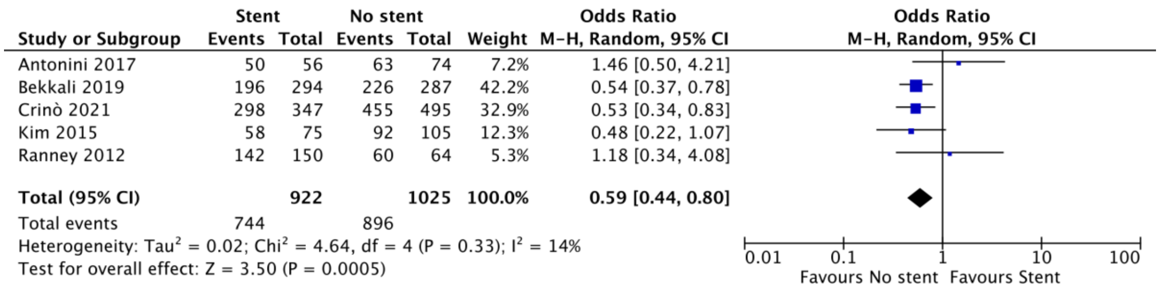

Supplementary Figure S2. Forest plot comparing diagnostic sensitivity in the subgroup of patients with a) plastic stent, and b) metal stent

a)

Study or Subgroup	Stent Events	Stent Total	No stent Events	No stent Total	Weight	Odds Ratio M-H, Random, 95% CI
Antonini 2017	50	56	63	74	15.2%	1.46 [0.50, 4.21]
Bekkali 2019	92	137	226	287	38.7%	0.55 [0.35, 0.87]
Crinò 2021	184	217	455	495	36.5%	0.49 [0.30, 0.80]
Ranney 2012	101	105	60	64	9.6%	1.68 [0.41, 6.98]
**Total (95% CI)**		**515**		**920**	**100.0%**	**0.68 [0.42, 1.10]**
Total events	427		804			

Heterogeneity: $Tau^2 = 0.10$; $Chi^2 = 5.48$, df = 3 (P = 0.14); $I^2 = 45\%$

Test for overall effect: Z = 1.56 (P = 0.12)

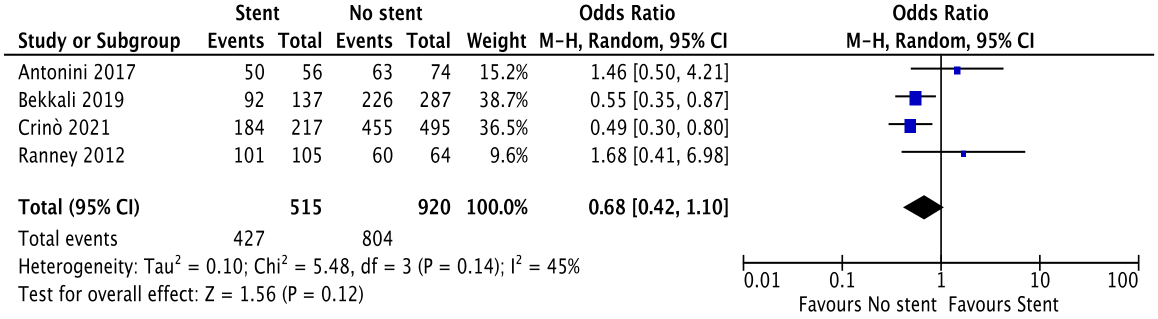

b)

Study or Subgroup	Stent Events	Stent Total	No stent Events	No stent Total	Weight	Odds Ratio M-H, Random, 95% CI
Bekkali 2019	104	157	226	287	65.7%	0.53 [0.34, 0.82]
Crinò 2021	116	130	455	495	30.2%	0.73 [0.38, 1.38]
Ranney 2012	43	45	60	64	4.1%	1.43 [0.25, 8.18]
**Total (95% CI)**		**332**		**846**	**100.0%**	**0.61 [0.43, 0.86]**
Total events	263		741			

Heterogeneity: $Tau^2 = 0.00$; $Chi^2 = 1.62$, df = 2 (P = 0.44); $I^2 = 0\%$

Test for overall effect: Z = 2.77 (P = 0.006)

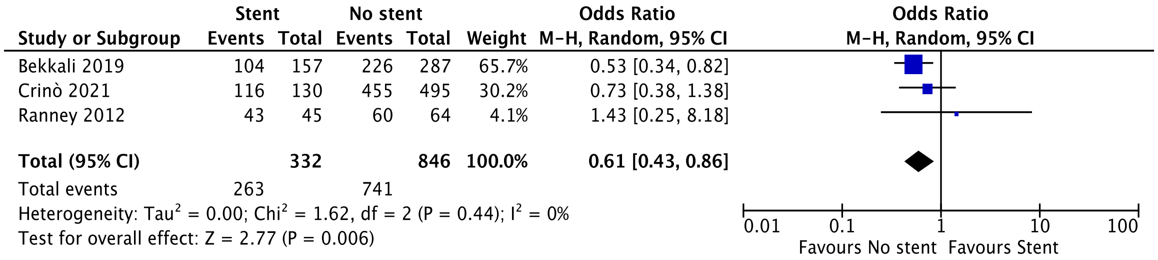

Supplementary Figure S3. Forest plot comparing the number of needle passes between patients with biliary stent versus patients without stent

	Stent			No stent				Mean Difference
Study or Subgroup	Mean	SD	Total	Mean	SD	Total	Weight	IV, Random, 95% CI
Antonini 2017	3.4	0.8	56	3.2	0.8	74	15.3%	0.20 [−0.08, 0.48]
Constantinescu 2022	2.03	0.48	68	2.13	0.63	175	19.2%	−0.10 [−0.25, 0.05]
Crinò 2021	2.98	0.79	347	2.81	0.68	495	20.2%	0.17 [0.07, 0.27]
Fisher 2011	4.3	1	98	4.5	1	170	16.2%	−0.20 [−0.45, 0.05]
Kim 2015	3	1.3	75	4	1.8	105	10.5%	−1.00 [−1.45, −0.55]
Ranney 2012	2	0.7	150	2	0.5	64	18.7%	0.00 [−0.17, 0.17]
**Total (95% CI)**			**794**			**1083**	**100.0%**	**−0.09 [−0.30, 0.11]**

Heterogeneity: $Tau^2 = 0.05$; $Chi^2 = 34.88$, df = 5 ($P < 0.00001$); $I^2 = 86\%$

Test for overall effect: Z = 0.87 (P = 0.38)

Mean Difference
IV, Random, 95% CI

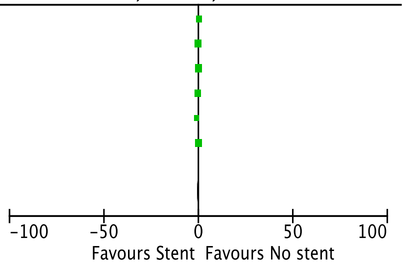

Supplement: Supplementary file 1 [file cancers-15-01789-s001.zip › cancers-2271925-supplementary.pdf]
